# The use of spleen biomechanics in forensic pathology

**DOI:** 10.1007/s00414-026-03857-3

**Published:** 2026-06-09

**Authors:** Johann Zwirner, Pavithran Devananthan, Natalia Kabaliuk, Paul D. Docherty, Benjamin Ondruschka

**Affiliations:** 1 Institute of Legal Medicine, University Medical Center Hamburg-Eppendorf, Hamburg, Germany; 2 Department of Oral Sciences, University of Otago, Dunedin, New Zealand; 3 Department of Mechanical Engineering, University of Canterbury, Christchurch, New Zealand; 4 Biomolecular Interaction Centre, University of Canterbury, Christchurch, New Zealand

**Keywords:** Biomechanical properties, Forensic tissue mechanics, Post-mortem interval, Spleen, Time since death estimation

## Abstract

Forensic tissue mechanics applies biomechanical analyses of human tissues to support forensic investigations, including time since death estimation. Unlike temperature-based methods such as the Henssge nomogram, which are limited once thermal equilibrium is reached, rheological tissue analyses remain applicable at extended post-mortem intervals (PMIs). While previous studies have demonstrated the utility of human brain and liver tissue mechanics, the forensic relevance of splenic biomechanics has not yet been systematically explored. In this study, rheological properties of post-mortem human spleen tissue were investigated to assess their applicability for PMI estimation and routine forensic diagnostics. Spleen samples from 53 adult cadavers (PMI range 42–518 h) and four pediatric cases (PMI range 63–112 h) were collected during forensic autopsies and analyzed using oscillatory shear rheometry after standardized storage at 4 °C. Storage modulus, loss modulus, and complex shear modulus were correlated with PMI, demographic variables, spleen weight, resuscitation status, and histological features of tissue. The loss modulus exhibited a significant negative correlation with PMI, whereas storage and complex shear modulus did not. Receiver operating characteristic analysis demonstrated that a loss modulus threshold of 333 Pa provided strong confirmatory evidence for PMIs shorter than 200 h (AUC = 0.831; positive likelihood ratio = 12.75). No significant associations were observed between rheological parameters and age at death, sex, spleen weight, resuscitation attempts, or histological findings, indicating that these factors are unlikely to confound PMI-related biomechanical changes. These findings identify the loss modulus of splenic tissue as a sensitive and robust biomechanical marker for PMI estimation even at extended intervals. Splenic tissue mechanics therefore represent a valuable addition to the multimodal framework of forensic tissue mechanics, particularly for extended intervals, complementing established analyses of other organs.

## Introduction

Forensic tissue mechanics describes the strategic biomechanical analysis of human tissues to obtain relevant information for criminal investigations [1]. With regard to time since death estimation, tissue mechanical analyses show promise beyond the post-mortem intervals (PMIs) covered by the routinely applied temperature-based Henssge method [2, 3]. The Henssge method is no longer applicable once body temperature and ambient temperature reach equilibrium, which depends on several factors, including the body mass index and the body surface area [4]. In contrast, rheological analyses of human brain tissue have proven useful for PMIs of up to 10 days at constant post-mortem ambient temperatures of 4 °C [5]. To date, brain [5] and liver tissues [6] are the only human organs that were analysed according to the principles of forensic tissue mechanics after the method had been established on ovine tissues [7–9]. A fundamental hypothesis of forensic tissue mechanics is that, under identical storage conditions, the biomechanical properties of each human organ change in a characteristic, time-dependent manner. Consequently, combining biomechanical properties from different organs is likely superior to analysing only a single organ per case when estimating time since death based on tissue mechanical analyses.

The spleen is an easily accessible and routinely examined parenchymatous organ at autopsy; however, to date it has been of minor forensic relevance [10]. Spleen palpation is a routine step during autopsy and enables the forensic pathologist to obtain observer-dependent, qualitative information about the biomechanical state of the organ. On palpation, a soft or “muddy” spleen with decreased firmness is a common sign of sepsis [11]. To date, only a few biomechanical studies have investigated post-mortem human spleen samples [12, 13]. Despite the routine examination of the spleen at autopsy, its biomechanical properties have never been systematically evaluated as a marker for time since death estimation or cause of death determination.

This study aims to perform a rheological analysis of post-mortem human spleen samples and to assess their applicability for time since death estimation and diagnostic questions relevant to routine forensic practice. To this end, the rheological properties of the spleen were assessed in a forensic autopsy cohort and related to PMI, age at death, spleen weight, sex, initiated resuscitation measures, and microscopic findings in spleen samples.

## Material/methods

### Tissue sampling and preparation

**Fig. 1:**
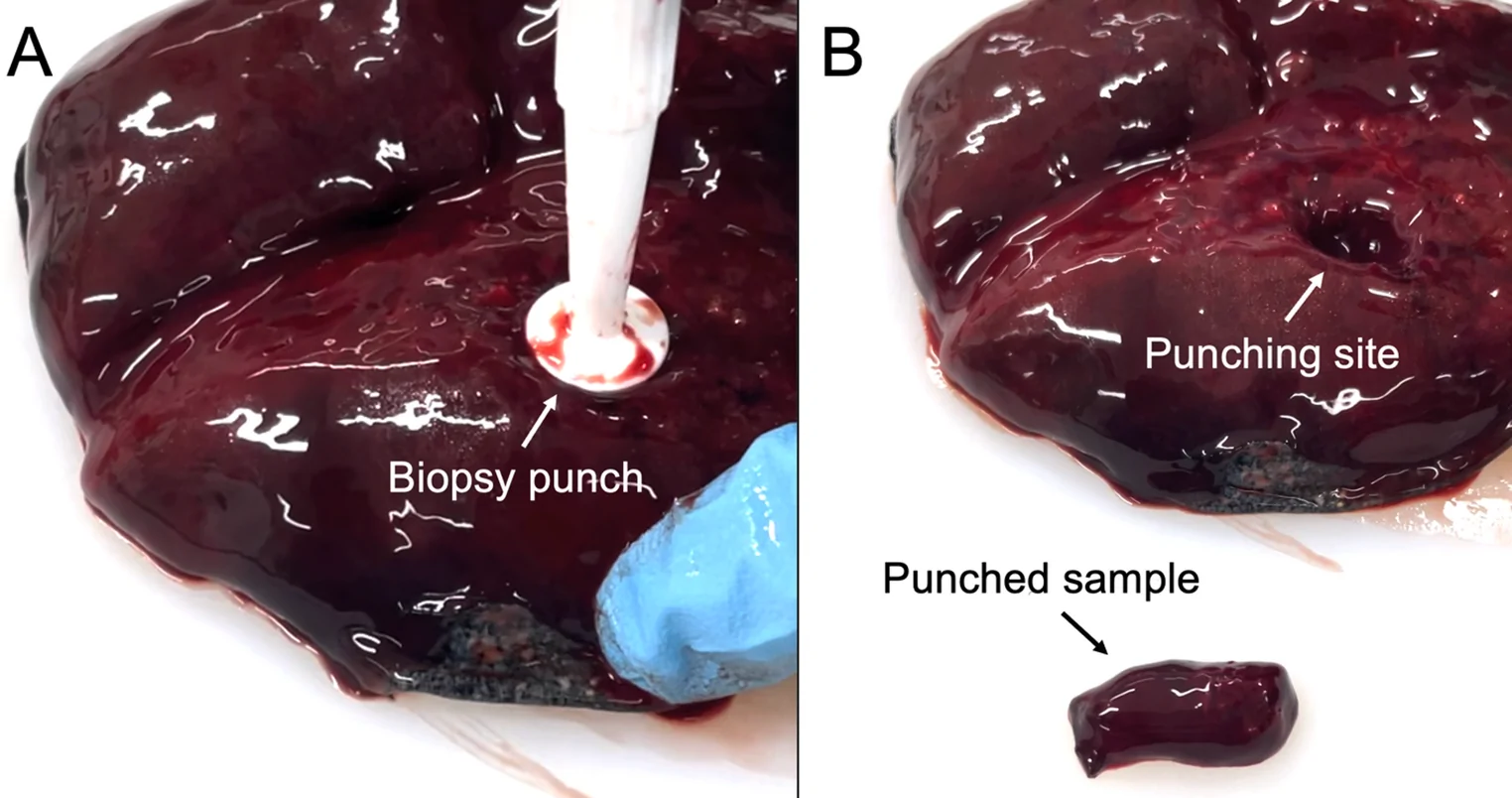
Representative images of the punching process are shown. (**A**) The sample is punched at the autopsy table from a rather muddy spleen with a commercial biopsy punch. (**B**) The punched sample is shown before height adaptation

Spleen samples from 53 adult cadavers (14 females, 39 males) were retrieved at the Institute of Legal Medicine, University Medical Center Hamburg-Eppendorf during forensic autopsies. The Ethics Committee of the Hamburg Medical Association (reference number: 2023-101023-BO-ff) approved the study. The ages at death of the cadavers ranged from 23 to 90 years with a mean (M) and standard deviation of 64 ± 16 years. The PMI was 208 ± 97 h, with a minimum and maximum of 42 and 518 h. The exact PMI was unknown in two of the mechanically analyzed cases. A rectal temperature of at least 30 °C upon admission to the cooling unit of the morgue and structural integrity of the organ at autopsy were defined as inclusion criteria. The spleen weight at autopsy was 192 ± 117 g with a range of 50 to 600 g. Additionally, spleen samples from four pediatric cases were tested. The age at death, cause of death, spleen weight, sex, and PMI for the pediatric cases are presented in the Results section (Table 3), together with the measured rheological properties. For the rheometer tests, circular cylindrical samples were punched from the splenic pulp using a commercial biopsy punch with a diameter of 10 mm; depth: 5 mm; Fig. 1). Following this, all samples were tested within two hours after the preparation and stored in sealed plastic containers at 4 °C until then.

## Biomechanical testing and histology

Biomechanical measurements were conducted using a rheometer (MCR302e; Anton Paar, Graz, Austria) operated with the RheoCompass software (Anton Paar). The spleen samples were positioned in the apparatus under an axial preload of 0.1 N to ensure full surface contact and adhesion. A 100-second relaxation period to allow residual stress dissipation was applied. For slippage minimization, waterproof sandpaper (Type P120, Federation of European Producers of Abrasives standard) was affixed to both the upper measuring plate and the base plate. Oscillatory shear tests were conducted with a peak angular shear strain of 3% at a frequency of 3 Hz. A continuous compressive force of 0.1 N over 50 cycles was applied. Phosphate-buffered saline (PBS) was used for rheometer calibration, and all measurements were performed in PBS at a controlled temperature of 20 °C. After the mechanical tests, all tested samples were put in 4% paraformaldehyde for full fixation and then histologically processed according to a standard protocol. For each sample, one H&E-stained slide was prepared in the circular plane of the tissue cylinder and jointly analysed by J.Z. and B.O. under a light microscope (Olympus BX51, Olympus Corporation, Tokyo, Japan) by consensus. The slides were screened for signs of inflammation (yes/no), blood congestion (yes/no) and fibrosis (no, mild, severe) upon gross inspection at 40x magnification. Representative images were scanned using a commercial software (PreciPoint, PreciPoint GmbH, Garching, Germany) and a digital microscope (M8; PreciPoint).

## Data compilation and analysis

The storage (G′), loss (G″), and complex shear moduli (G*) were determined for all samples. Data were entered into Microsoft Excel, version 16.74 (Microsoft Corporation, Redmond, USA), and then processed and analyzed using GraphPad Prism, version 9 (GraphPad Software, La Jolla, USA). The Shapiro-Wilk test was applied to evaluate the normality of data distributions. The measured rheological properties were correlated with age at death, PMI and the spleen weight measured at autopsy. Depending on the distribution, either Pearson’s (P(r)), for normal distribution, or Spearman’s (S(r)), for non-normal distribution, correlation coefficients were calculated. Group comparisons of the measured rheological spleen properties were performed for males vs. females, cadavers with vs. without cardiopulmonary resuscitation based on autopsy reports, cadavers without microscopic signs of liver pathology vs. fatty livers, with microscopic signs of inflammation vs. non-inflamed samples, with microscopic signs of fibrosis vs. non-fibrous samples and samples with microscopic blood congestion signs vs. uncongested samples. In addition, the spleen weight, age at death and PMI was compared for all of the abovementioned groups to exclude any bias based on one of the three factors. For group comparisons, either unpaired t-tests with Welch’s correction or unpaired Mann-Whitney U-tests were performed. P-values ≤ 0.05 were considered statistically significant.

## Results

### Post-mortem interval

**Fig. 2:**
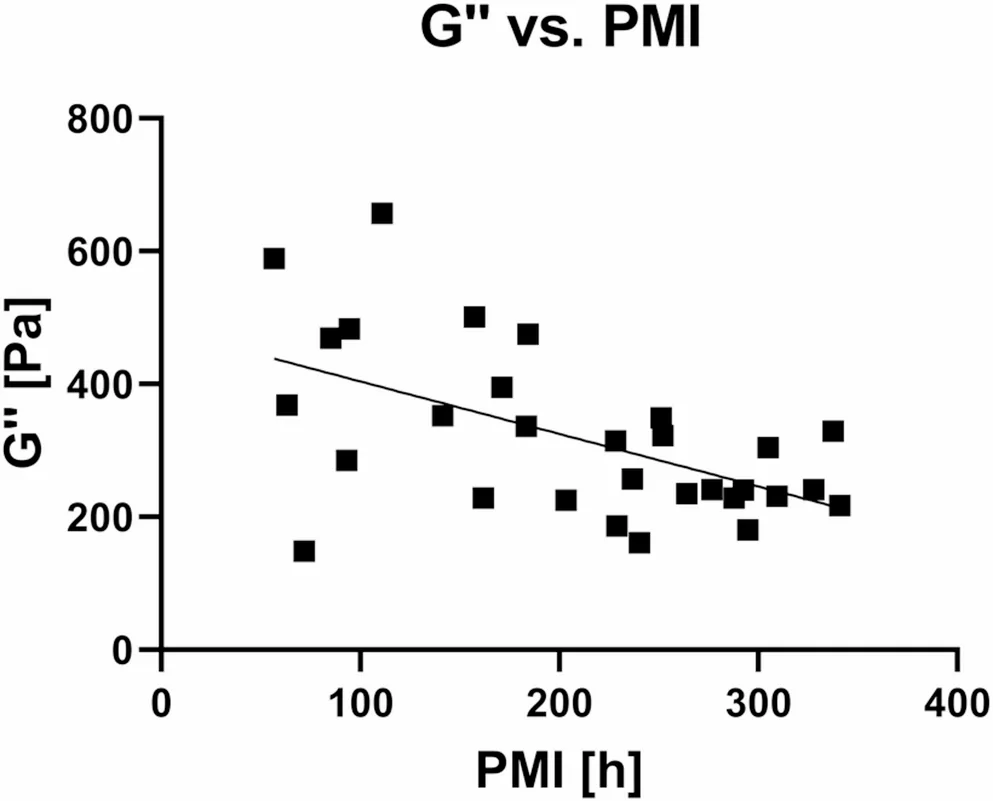
The significant negative correlation between the loss modulus (G’’) and the post-mortem (PMI) interval is shown

**Fig. 3:**
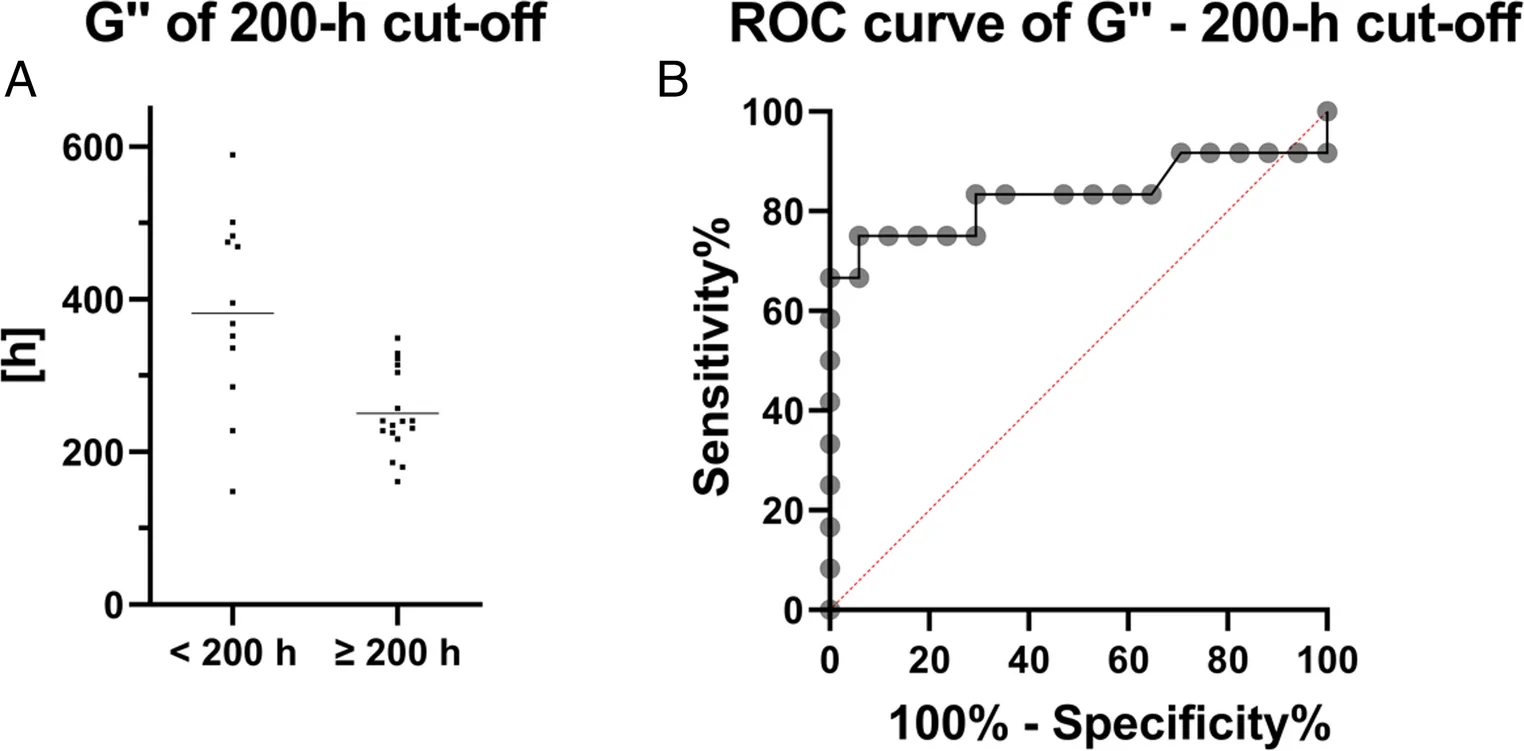
The data for the 200-h cut-off of the loss modulus (G’’) is depicted. (**A**) The G’’ values are shown as a scatter plot with the horizontal line representing the median. Values above median are only reached below 200 h. (**B**) The Receiver Operating Characteristic (ROC) curve is depicted

Samples from 32 of the 53 adult spleens (60%) and all of the pediatric spleens could be punched. All punched samples could be biomechanically tested. The G’’ showed a significant negative correlation with the PMI (P(r) = -0.551; *p* = 0.002; Fig. 2). G’ (P(r) = -0.238, *p* = 0.200) and G* (P(r) = -0.286, *p* = 0.130) were not significantly correlated with the PMI. Hence, Receiver Operating Characteristic (ROC) curves were calculated only for the G’’ in 50-hour steps for PMI thresholds of 100 to 300 h. For the G’’ cut-off value with the highest calculated positive likelihood ratio, the results are depicted in Table 1 for PMI sub-group. The data of the 200-h group is depicted in detail in Fig. 3 as this was the PMI threshold with the highest positive likelihood ratio of all investigated sub-groups.

**Table 1:** The results of the Receiver Operating Characteristic curves are depicted for the loss modulus, which revealed a significant negative correlation with the post-mortem interval (PMI). The PMI was divided into steps of 50 h. Only the highest calculated positive likelihood ratio (LR+) is depicted. AUC, area under the curve; CI, confidence interval; Se, sensitivity; Sp, specificity

PMI < [h]	AUC	Cut-off value [Pa]	Se [%]	95% CI [%]	Sp [%]	95% CI [%]	LR+
100	0.717	> 479	33	5.9–70.0	96	79.0–99.8	7.67
150	0.773	> 479	38	13.7–69.4	95	78.2–99.8	8.25
200	0.831	> 333	75	46.8–91.1	94	73.0–99.7	12.75
250	0.692	> 333	56	33.7–75.4	92	64.6–99.6	6.67
300	0.613	> 309	50	31.3–68.6	80	37.6–99.0	2.50

### Age at death, spleen weight, sex, and cardiac resuscitation

None of the measured rheological properties was significantly correlated with age (G’: P(r) = -0.112; *p* = 0.540; G’’: S(r) = -0.272; *p* = 0.130; G*: P(r) = -0.141; *p* = 0.440) or spleen weight at autopsy (G’: P(r) = -0.178; *p* = 0.920; G’’: S(r) = -0.156; *p* = 0.400; G*: P(r) = -0.069; *p* = 0.710). None of the rheological properties differed significantly between males and females (Welch’s test; G’: t(10.39) = 0.59, *p* = 0.568; G’’: t(20.66) = 0.99, *p* = 0.334; G*: t(10.60) = 0.47, *p* = 0.651) and whether a cardiac resuscitation had been performed (Welch’s test; G’: t(26.49) = 0.51, *p* = 0.613; G’’: t(28.23) = 0.95, *p* = 0.348; G*: t(27.01) = 0.35, *p* = 0.732). An overview of the age at death, spleen weight, sex and PMI as well as the mean G’, G’’ and G* values of all here tested sub-groups is given in Table 2.

**Table 2:** The means of the age at death, spleen weight, sex ratio, post-mortem interval (PMI), storage modulus (G’), loss modulus (G’’) and complex shear modulus (G*) of all investigated sub-groups in this study are depicted

Group	Age at death [y]	Spleen weight [g]	Sex [f: m]	Samples	PMI [h]	G’ [Pa]	G’’ [Pa]	G* [Pa]
Females	71	123	-	14	214	1148	294	1187
Males	58	200	-	39	206	1053	337	1110
Mechanics group	61	175	9:23	32	209	1080	325	1131
No mechanics group	67	213	5:16	21	209	-	-	-
With inflammation	68	244	2:4	6	282	881	219	908
Without inflammation	60	165	6:16	22	184	1115	350	1182
Mild fibrosis	59	223	2:18	20	220	1044	289	1086
Severe fibrosis	65	106	3:3	6	189	1019	391	1121
With blood congestion	63	190	5:12	17	194	1128	347	1198
Without blood congestion	60	169	3:8	11	224	967	284	1010
With resuscitation	60	186	3:11	14	233	1111	315	1158
Without resuscitation	64	161	5:11	16	177	1052	343	1110

## Histology-based mechanical comparisons

**Fig. 4:**
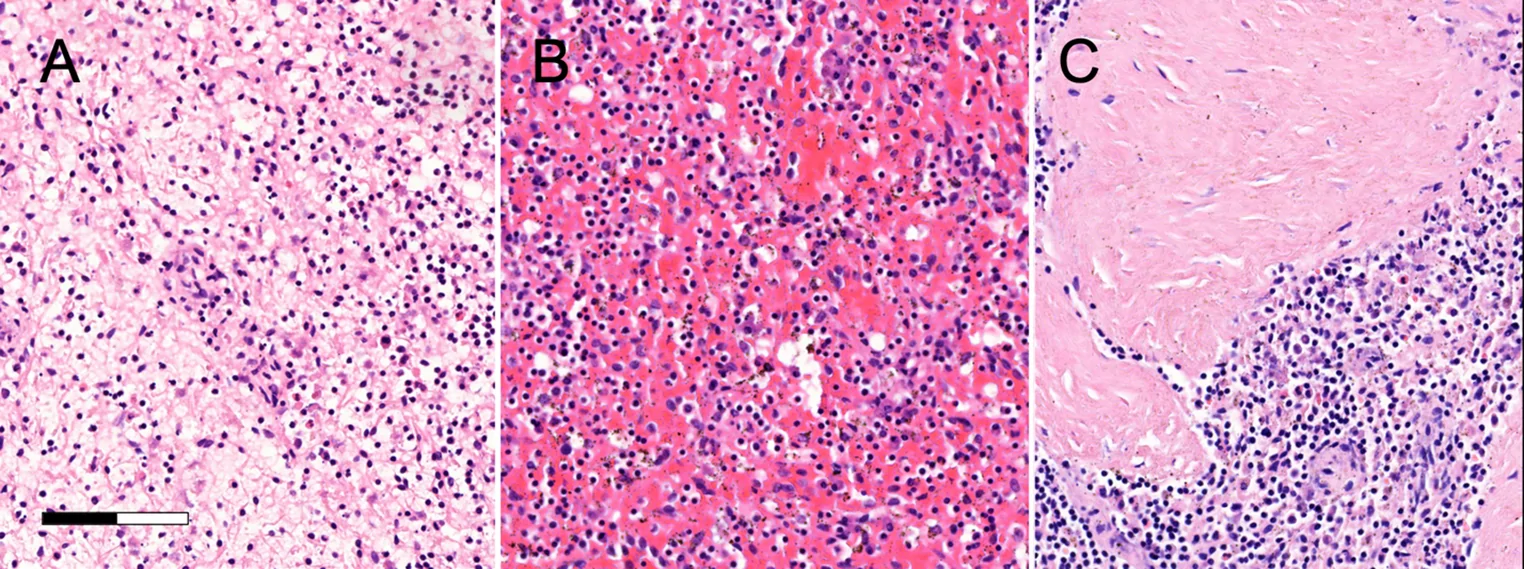
Representative images of the microscopic investigation of the tested spleen samples are shown. (**A**) Blood congestion is absent. (**B**) The pulp is congested with erythrocytes. (**C**) Fibrous tissue can be detected

G’ of spleen samples with microscopic signs of inflammation was not significantly different from samples without inflammatory cells (Welch’s test; t(15.50) = 2.12, *p* = 0.051), however, they showed significant differences in regard to G’’ (Welch’s test; t(25.76) = 4.08, *p* < 0.001) and G* (Welch’s test; t(16.64) = 2.39, *p* = 0.029). Neither of the measured rheological properties differed significantly between samples with mild and severe fibrosis (Welch’s test; G’: (t(19.75) = 0.24, *p* = 0.811); G’’: (t(6.29) = 1.41, *p* = 0.206); G*: (t(12.51) = 0.29, *p* = 0.775). The rheological properties of samples with and without blood congestion were not significantly different (Welch’s test; G’: (t(25.96) = 1.34, *p* = 0.191); G’’: U = 67.5, *p* = 0.230; G*: (t(24.99) = 1.45, *p* = 0.160). Representative images of the microscopic investigation are shown in Fig. 4.

## Spleen weight, age and PMI of punching fails and mechanically compared groups

The samples that could not be punched and, therefore, not mechanically tested were not significantly different from the tested samples in regard to spleen weight (Mann-Whitney U-test; U = 246, *p* = 0.101), age at death (Welch’s test; t(50.29) = 1.33, *p* = 0.189) and PMI (Welch’s test; t(29.84) = 0.01, *p* = 0.992). Spleen samples with histological signs of inflammation had a significantly higher PMI compared to samples without signs of inflammation (Welch’s test; t(15.82) = 3.68, *p* = 0.002). The female age at death was significantly lower compared to the male age at death (Welch’s test; t(22.12) = 2.40, *p* = 0.026). The samples with mild microscopic signs of fibrosis were significantly lighter when compared to the samples with severe signs of fibrosis (Mann-Whitney U-test; U = 21, *p* = 0.024).

### Pediatric cases

An overview of the measured rheological properties of the four pediatric cases as well as the age at death, the cause of death, spleen weight, sex and PMI is given in Table 3.

**Table 3:** The age, cause of death (COD), spleen weight, sex, post-mortem interval (PMI), the storage modulus (G’), the loss modulus (G’’) and the complex shear modulus (G*) are depicted for the investigated pediatric cases

Age [y]	COD	Spleen weight [g]	Sex	PMI [h]	G’ [Pa]	G’’ [Pa]	G* [Pa]
0.02	Heart failure	10	f	63	1453	338	1491
2	Sepsis	41	m	64	2171	451	2217
5	TBI	64	m	112	1366	443	1436
6	Polytrauma	99	m	108	1771	890	1982

## Discussion

The spleen generally plays a limited role in forensic diagnostics, except in cases of blunt splenic trauma, treatment-related injury, anaphylaxis, drowning, and sepsis [10]. This study focused on the post-mortem tissue mechanical characteristics of the human spleen, with particular emphasis on changes related to the PMI as well as factors derived from police records and autopsy findings. The aim was to explore the applicability of spleen biomechanics in routine forensic investigations and to expand the evidentiary value that can be derived from this organ.

### The loss modulus of splenic tissue is a useful PMI marker

Within the investigated PMI range of 42 to 518 h, the G’’ was the only one of the three measured rheological parameters that showed a significant change. From a tissue mechanical perspective, G’’ reflects processes associated with energy dissipation during deformation [14]. For example, post-mortem dehydration and fluid redistribution result in reduced viscous resistance to deformation and, consequently, a decrease in G’’. In contrast, the G’ reflects the ability of tissue to store energy [14]. Energy storage is predominantly related to tissue architecture, including collagen and elastin networks. The absence of significant changes in G’ values within the investigated PMI range indicates that there is no biomechanical evidence of autolytic degradation of the splenic extracellular matrix during this period.

Based on receiver operating characteristic (ROC) curve analysis, G’’ values above 333 Pa can be used as a confirmatory threshold for PMIs of less than 200 h at 4 °C. Given a positive likelihood ratio of 12.75, this threshold constitutes strong diagnostic evidence. Compared with previously reported data for brain tissue [5], G’’ analysis of splenic tissue stored at 4 °C demonstrated superior confirmatory power for the 200 h threshold. In contrast, rheological analysis of brain tissue within a PMI range of 42 to 341 h showed optimal performance at thresholds of 150 and 250 h. Taken together, these findings demonstrate for the first time how biomechanical analyses of different tissue types may complement each other within the framework of forensic tissue mechanics.

In addition to its role in time since death estimation, the PMI must be considered a relevant confounding factor. Samples exhibiting microscopic signs of inflammation showed significantly lower G’’ and G* values compared with samples without inflammatory changes. However, the significantly longer PMI observed in samples with microscopic inflammation indicates that the observed differences were likely confounded by PMI rather than being directly attributable to inflammation.

Beyond its utility for time since death estimation, the present study demonstrates that PMI is a key factor influencing the biomechanical properties of splenic tissue more than other case variables. Accordingly, post-mortem intervals until autopsy should be kept as short as possible to preserve the biomechanical behavior of splenic samples. Within the PMI range of 42 to 518 h analyzed in this study, a marked decrease in G’’ values was observed beyond approximately 200 h post-mortem. Notably, the earliest investigated post-mortem time point was 42 h. In principle, biomechanical changes may begin immediately after death and progress during the first 42 h prior to the earliest data acquisition in this study. To date, no published biomechanical investigations have demonstrated significant changes in splenic rheometric properties within the first 42 h post-mortem at 4 °C. Kemper et al. reported that human splenic samples were tested within 48 h post-mortem to minimize adverse effects of tissue degradation; however, no correlation between biomechanical properties and PMI was performed [12]. Stingl et al. investigated critical tension and elastic modulus in 21 spleens with PMIs ranging from 24 to 36 h [13]. Due to the limited number of successfully tested samples, no statistical analysis was performed, and the relationship between biomechanical properties and PMI was not further addressed. In contrast, Taskent et al. investigated splenic stiffness in male Sprague-Dawley rats up to 36 h post-mortem at approximately 23 °C using shear-wave elastography and observed a progressive decline in stiffness over the analyzed PMI range [15]. At ambient temperatures of around 20 °C, it is anticipated that the PMI-related changes in G’’ detected here would occur sooner after death, as rheometric properties are known to be temperature-sensitive [9].

### Several key parameters are unlikely to confound PMI-related rheological changes

Based on the results of this study, there is no evidence that the G’, G’’, or G* of the spleen depend on age at death, spleen weight, sex, cardiac resuscitation attempts, and microscopic organ features. Moreover, no significant difference was detected for microscopically congested and uncongested samples. One clear limitation of splenic tissue analysis is that samples could be prepared from only approximately 60% of spleens using the punching method applied in this study. This likely reflects advanced autolytic degradation or enzymatic destruction of splenic pulp leading to insufficient structural integrity for cylindrical sampling. In comparison, 95% of brain samples [5] and all liver samples [6] obtained at autopsy could be prepared and subsequently subjected to biomechanical testing. In practical terms, failure to prepare a splenic sample meant that it was not possible to punch a specimen that sufficiently retained its shape for loading into the testing apparatus using a biopsy punch. In routine practice, the number of available spleen samples may be further reduced because some individuals have previously undergone splenectomy, as the spleen - unlike the brain or liver - is a non-vital organ. In cases of splenic injury, it may nevertheless still be possible to punch a sample from the remaining (and ‘intact’) tissue, as the required test specimen is small relative to the size of the entire organ. The reasons why a substantial proportion of splenic samples could not be successfully punched are beyond the scope of this manuscript. However, no significant differences were detected between unsuccessful punching attempts and successfully tested samples with respect to age at death, spleen weight, or PMI. The number of unsuccessful punching attempts may be reduced in samples with PMIs shorter than 42 h, which represents the earliest time point investigated in this study.

### Splenic tissue mechanics in literature and its value beyond forensic practice

Beyond forensic applications, there is a need for comprehensive characterization of the mechanical properties of splenic tissue for both basic research and clinical practice. High-quality biomechanical data on the spleen are essential for finite element modelling of blunt force injuries [16] and for the development of safety gear [17]. To date, post-mortem biomechanical properties of the spleen have been investigated predominantly in animal models [18–23], with only a few studies employing human tissue samples [12, 13].

In addition, biomechanical properties of the spleen are required for the implementation and refinement of haptic feedback for internal organs in surgical training simulators [24]. Beyond modelling and simulation, non-invasive measurements of splenic stiffness appear to be superior to liver stiffness measurements for monitoring portal hypertension in patients with advanced chronic liver disease [25, 26] and may serve as a predictor of survival following portosystemic shunt implantation [27].

The generally comparable viscoelastic properties suggest that, despite age-related differences in tissue composition and size, the mechanical response of pediatric spleen tissue falls within the range observed in adult samples. However, due to the small number of pediatric cases, no statistical testing was performed, and the observations are introduced as exploratory.

Notably, the spleens investigated in the present study were retrieved during autopsy and tested within two hours thereafter. This approach reflects the most common scenario in routine forensic practice, in which a body is discovered intact with an unknown time of death. From a practical standpoint, the scenario of an isolated human spleen being recovered after removal by a perpetrator remains largely theoretical. In routine casework, isolated internal organs presented for forensic evaluation are typically slaughter by-products submitted by laypersons for clarification of potential human origin, most frequently involving the heart.

### Future research

In this study, human spleen samples with a mean PMI of approximately nine days stored at 4 °C were analyzed. The applied method was shown to be temperature sensitive in the animal model [9]. Hence, future investigations should investigate other forensically relevant storage temperatures including around 20 °C (room temperature) and 37 °C (body temperature). Moreover, rheometric data should be collected immediately post-mortem to gain “point zero” reference values and establish a baseline for the post-mortem changes.

## Conclusions

The G’’ of human splenic tissue is a sensitive biomechanical marker for time since death estimation. A G’’ threshold of 333 Pa provided strong confirmatory evidence for PMIs of less than 200 h. In the here investigated sample, there was no evidence that the G’, G’’, or G* of the spleen depend on age at death, spleen weight, sex, cardiac resuscitation attempts, microscopic changes of the spleen and macroscopic signs of chronic splenic congestion.

### Limitations

The study was limited in sample size. Unequal group sizes reduced power for detecting omnibus effects. The high within-group variance of the data, which is common when analyzing biological samples, likely contributed to the non-significant results, especially when small to moderate effect sizes were detected. The rheological changes before 42 h remain unclear as this was the earliest time post-mortem that a sample was investigated.
